# Metabolic Changes in Patients with Premature Ovarian Insufficiency: Adipose Tissue Focus—A Narrative Review

**DOI:** 10.3390/metabo15040242

**Published:** 2025-04-02

**Authors:** Miriam Sánchez-García, Kapy León-Wu, Regina de Miguel-Ibáñez, Nitzia López-Juárez, Claudia Ramírez-Rentería, Etual Espinosa-Cárdenas, Ernesto Sosa-Eroza, Manuel R. García-Sáenz

**Affiliations:** 1 Hospital Angeles Culiacán, Policlinics, Culiacán Sinaloa 80100, Mexico; mrmsaga@gmail.com; 2 Endocrinology Service, Hospital de Especialidades del Centro Médico Nacional Siglo XXI, Instituto Mexicano del Seguro Social, Mexico City 06720, Mexico; kapy_l_w@outlook.com (K.L.-W.); espinosaetual@gmail.com (E.E.-C.); esosa@yahoo.com (E.S.-E.); 3 Hospital Angeles del Pedregal, Ciudad de México 10700, Mexico; reginadm94@gmail.com; 4 Endocrinology Service, Hospital de Cardiología del Centro Médico Nacional Siglo XXI, Instituto Mexicano del Seguro Social, Mexico City 06720, Mexico; nitglj@gmail.com; 5 Unidad de Investigación en Enfermedades Endocrinas, Hospital de Especialidades del Centro Médico Nacional Siglo XXI, Instituto Mexicano del Seguro Social, Mexico City 06720, Mexico; cramirez@endocrinologia.org.mx

**Keywords:** primary ovarian insufficiency, estrogens, adipose tissue, lipid metabolism

## Abstract

**Background:** Estrogen plays a crucial role in adipose tissue homeostasis, influencing fat distribution, lipid metabolism, and insulin sensitivity. Through estrogen receptor (ER) activation, particularly ERα, estradiol (E2) regulates adipogenesis, inhibits adipocyte hypertrophy, and promotes insulin signaling. It enhances lipid oxidation, reduces lipogenesis, and suppresses pro-inflammatory cytokine production, thereby maintaining metabolic health. Primary ovarian insufficiency (POI), characterized by estrogen deficiency before the age of 40, disrupts this regulatory network, leading to adverse metabolic effects. **Objetives:** This review examines the effects of estrogen on adipose tissue, lipid metabolism, and carbohydrate metabolism, with a particular focus on clinical evidence in women with POI. Methods: A narrative review of the metabolic alterations associated with POI, emphasizing the molecular, biochemical, and metabolic mechanisms underlying estrogen deficiency, with a special focus on adipose tissue. **Results:** Women with POI exhibit increased visceral fat accumulation, reduced lean mass, and alterations in adipokine secretion, resembling the metabolic phenotype of postmenopausal women. The decline in estrogen levels contributes to central adiposity, impaired lipid metabolism, and insulin resistance, exacerbating the risk of type 2 diabetes (T2D) and cardiovascular disease (CVD). The loss of estrogenic regulation leads to enhanced lipolysis in visceral fat, raising free fatty acid flux to the liver, promoting hepatic steatosis, and worsening insulin resistance. Studies indicate that POI patients have significantly higher total cholesterol, low-density lipoprotein (LDL) cholesterol, and triglycerides compared to age-matched controls, reinforcing their heightened CVD risk. Reduced sex hormone-binding globulin (SHBG) levels increase free androgen availability, aggravating central fat deposition. These metabolic disturbances can potentially accelerate atherosclerosis and vascular aging, increasing morbidity and mortality in POI patients. **Conclusions:** Understanding the role of estrogen in adipose tissue and its disruption in POI highlights the importance of early intervention. Although the available evidence is limited and largely extrapolated from menopause studies, strategies such as hormone replacement therapy, lifestyle modifications, and lipid profile optimization are essential to mitigate metabolic consequences and improve long-term health outcomes in women with POI.

## 1. Introduction

Historically, estrogenic compounds were defined as substances capable of stimulating uterine growth and increasing progesterone receptor synthesis. However, estrogens are now recognized for their pleiotropic effects, exerting biological actions in multiple systems, including the cardiovascular system, brain, bone, liver, adipose tissue, colon, skin, and salivary glands, among others [1,2]. In pharmacology, their ability to act as both agonist and antagonist activity on estrogen receptors (ERs) has led to diverse applications, such as the development of contraceptives, postmenopausal hormone replacement therapy, treatments for breast cancer, and osteoporosis management. Furthermore, ERs play a key role in various diseases, breast and endometrial carcinomas, autoimmune disorders, and, notably, cardiovascular risk [1].

Adipose tissue expresses ERs, highlighting the regulatory role of estrogens in adipocyte function. Specifically, ERα is primarily involved in adipocyte growth and proliferation, while ERβ influences the sex-specific distribution of adipose tissue [3]. The sexual dimorphism in fat distribution is largely determined by sex steroids and the differential expression of ERs. Males tend to accumulate more visceral adiposity (“apple-shaped” fat distribution), whereas females predominantly store fat subcutaneously (“pear-shape” fat distribution). The distribution pattern has important metabolic implications, as the higher subcutaneous-to-visceral fat ratio in premenopausal women is associated with a lower risk of cardiometabolic diseases. In contrast, decreased circulating estrogen levels are linked to an increased risk of obesity, T2D, and CVD. Supporting this, E2 replacement therapy has been shown to reduce visceral adiposity and, consequently, CVD risk [4].

Estrogens also play a critical role in lipid metabolism. They reduce LDL cholesterol and increase high-density lipoprotein (HDL) cholesterol by upregulating hepatic LDL receptor expression, which enhances the plasma LDL clearance and promotes cholesterol excretion into bile. Additionally, estrogens decrease lipase activity, leading to lower total cholesterol concentrations while increasing HDL levels, thereby contributing to a favorable lipide profile [5]. Beyond lipid metabolism, estrogens influence insulin sensitivity through direct actions on insulin-sensitive tissues and indirect mechanisms, such as modulation of oxidative stress. In skeletal muscle, ERα upregulates GLUT4 expression, enhancing glucose uptake. At the central level, estrogen modulates appetite regulation in the hypothalamus, and estrogen deficiency is associated with increased appetite and reduced insulin sensitivity, further contributing to metabolic dysregulation [6].

In the context of POI—a condition characterized by reduced ovarian function and decreased estrogen levels—the absence of estrogen leads to metabolic alterations similar to those observed in postmenopausal women. This deficiency is associated with changes in fat distribution, as well as disruptions in lipid and carbohydrate metabolism, contributing to an increased cardiometabolic risk in affected individuals.

In this narrative review, we examine the effects of estrogen on adipose tissue, lipid metabolism, and carbohydrate metabolism, with a particular focus on clinical evidence in women with POI. We begin by outlining the fundamental aspects of estrogen structure and receptor mechanisms, followed by an analysis of its role in adipose tissue and metabolic regulation. Finally, we discuss the consequences of estrogen deficiency in women with POI.

## 2. Methods

We conducted a narrative review using the literature up to June 2024 in the PubMed search engine, analyzing full-text articles or summary articles available in English that included information on the influence of estrogens on adipose tissue, lipid metabolism, carbohydrate metabolism, and the physical and metabolic changes in patients with menopause and POI. We focused on the associated metabolic alterations, emphasizing the molecular, biochemical, and metabolic mechanisms to enhance the understanding of estrogen deficiency in these women and highlight the importance of treatment.

## 3. Estrogen and Estrogen Receptors

**Figure 1:**
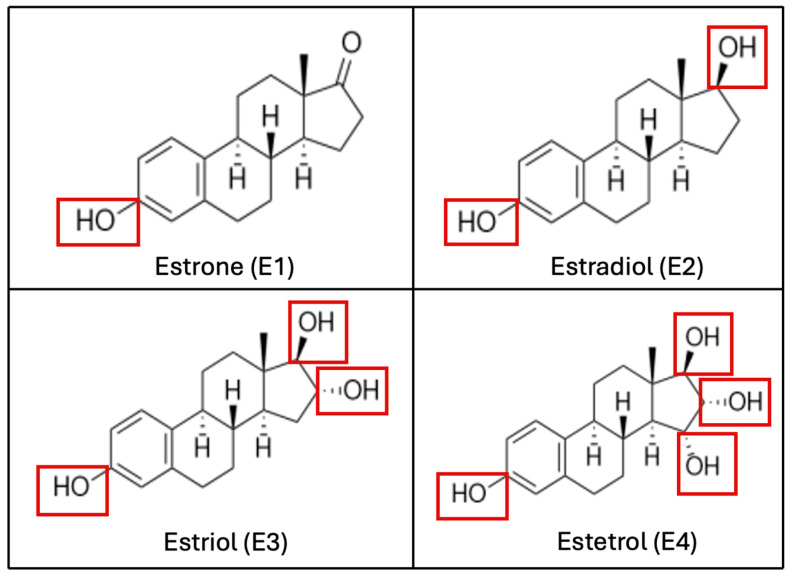
Biochemical structure of estrogenic compounds. The number of hydroxylations, which determines the compound’s name, is highlighted in red. (**E1**) is the predominant estrogen during menopause, (**E2**) is the main estrogen in reproductive-age women, (**E3**) is primarily produced during pregnancy, and (**E4**) is exclusively synthesized during the fetal stage.

Endogenous natural estrogens include E2, with its two isoforms 17-αE2 and 17-βE2, estrone (E1), estriol (E3), and estetrol (E4). They share a common structure of four rings (A, B, C, D), a hydroxyl group at C3, and either a hydroxyl or ketone group at C17 (see Figure 1). Structural characteristics are critical, as the phenolic A-ring facilitates high-affinity binding to the ER [7,8,9].

E2 is primarily synthesized by the ovaries, while E1 and E3 are produced in the liver from E2 or in peripheral tissues from androstenedione (A4). Most circulating estrogen is tightly but reversibly bound to SHBG. A small proportion (approximately 2–3%) remains free and active, rapidly distributing due to its small size and lipophilic nature. E2 has a half-life of approximately three hours and undergoes dynamic interconversion with E1 and E3 [2,7].

Throughout a woman’s reproductive life, circulating estrogen levels fluctuate. E2 is the most potent and predominant estrogen from menarche to the premenopausal stage. In terms of hormonal activity, E2 is approximately ten times more potent than E1 and about 100 times more potent than E3 and E4. In the postmenopausal stage, E1, synthesized in adipose tissue from adrenal dehydroepiandrosterone (DHEA), replaces E2 as the predominant estrogen [10]. During pregnancy, placental production significantly increases E3 levels, making it the primary circulating estrogen. E3 increases progressively throughout pregnancy, peaking at term, with 90% being excreted in urine [11]. E4 is uniquely synthesized in the fetal liver and is only detectable during pregnancy. Outside pregnancy, E3 and E4 levels are minimal in non-pregnant women [12]. Estrogens are synthesized from various precursors, with aromatase (CYP19A1) being the key enzyme for C18 estrogen synthesis. Aromatase is expressed in multiple tissues, including granulosa cells, bone, skin, brain, endometrium, and adipose tissue. In the ovaries, testosterone serves as the primary precursor for E2 synthesis via aromatase. Conversely, in adipose tissue, A4, derived from DHEA and its sulfated form (DHEA-S), is converted to E1 by aromatase. Steroid production in adipose tissue varies based on a woman’s reproductive stage (increasing in menopause) and nutritional status [8].

For estrogens to exert their effects, they must bind to ERs. ERα (also known as ER1 or ESR1) has at least three isoforms and is encoded on chromosome 6q25.1, while ERβ (also known ER2 or ESR2) has at least five isoforms and is encoded on chromosome 14q23.2. Both are ligand-activated transcription factors, leading to relatively slow biological responses [1,3]. The discovery of rapid, non-genomic estrogen effects led to the identification of the G protein-coupled estrogen receptor (GPER or GPR30). Additionally, ERα and ERβ have been detected in the plasma membrane [13].

Among the three ERs, ERα deletion results in the most significant disruptions [14]. ERα knockout models exhibit infertility due to hypothalamic–pituitary–gonadal axis dysregulation [15]. Mammary duct development is impaired, obesity and insulin resistance occur, vascular protective effects of estrogen are lost, lupus susceptibility increases, pancreatic beta-cell viability decreases, and cortical bone mineral density is reduced, delaying epiphyseal closure after puberty [16,17,18,19,20,21]. ERα is primarily expressed in the uterus, breast, liver, kidney, adipose tissue, ovaries, bone, brain, and, in males, in the epididymis, prostate, and testes [2].

ERβ deletion results in subtler phenotypic changes. ERβ knockout models exhibit reduced fertility and fewer offspring due to impaired ovulation and reduced LH surges, as ERβ is expressed in granulosa cells. Thus, ERβ is essential for folliculogenesis, ovarian function, and ovulation, potentially maintaining granulosa cell differentiation [14]. ERβ is found in the colon, salivary glands, endothelium, lungs, bladder, ovaries, bone, brain, and, in males, the prostate and testes [2].

GPER deletion does not cause infertility but appears to have a metabolic role, promoting obesity and glucose intolerance [22]. Notably, certain drugs, including fulvestrant, tamoxifen, and raloxifene, act as GPER agonists [14]. GPER is expressed in the central and peripheral nervous system, uterus, ovaries, breasts, testes, gastrointestinal system, pancreas, kidneys, liver, adrenal glands, pituitary, bone, cardiovascular system, and immune cells [2].

ER signaling mechanisms include direct (classical) estrogen response element (ERE) binding, indirect (gene regulation without EREs binding), non-genomic pathways (e.g., GPER), and ligand-independent activation [2]. Beyond endogenous estrogens, metabolites, endogenous compounds (e.g., 27-hydroxycholesterol), and exogenous substances (e.g., phytoestrogens, genistein, daidzein, equol) can also bind ERs [1].

## 4. Regulation of Adipogenesis and Fat Distribution by Estrogens

Estrogens regulate the key steps of preadipocyte differentiation, proliferation, and both white and brown adipogenesis [23]. They influence adipogenesis through various cellular and molecular mechanisms. Research by Saavedra-Peña et al., indicates that, in mice, adipocyte progenitor cells (APCs) respond differently to estrogen signaling, which regulates their proliferation and differentiation [4,24]. Notably, ERα plays a significant role in controlling the fate of APCs; its loss leads to lipodystrophy, indicating a switch from adipogenic to myofibrotic cell lineages [25]. The distinct responses of APCs in subcutaneous and visceral depots to estrogen further elucidate the complex relationship between sex steroids and fat distribution [4].

Estrogens, produced in adipocytes via aromatization from androgenic precursors, increase in proportion to total body adiposity. ERα is expressed in adipose tissue, and its deletion in mice leads to increased adiposity, particularly in visceral adipose depots. This increase is observed in both sexes, contrasting ERβ knockout mice, which do not become obese, indicating ERα’s more significant role in preventing fat deposition and regulating adipocyte proliferation [4,23].

Heine et al. demonstrated that mice with a global ERα-knockout (ERαKO), regardless of sex, exhibit a significant increase (50–180%) in adipocyte number, accompanied by insulin resistance, glucose intolerance, and liver steatosis [26]. A similar phenotype was observed by Jones et al. in aromatase knockout (ArKO) mice, which lack endogenous estrogens synthesis, as well as in patients with mutations in CYP19A1 and ESR1 [27]. The primary mechanism by which estrogens influence adipocyte proliferation appears to involve the inhibition of peroxisome proliferator-activated receptor gamma (PPARγ) coactivator recruitment, including steroid receptor coactivator-1 (SRC-1) and cyclic adenine monophosphate (cAMP) response element binding protein (CREB)-binding protein (CBP). Other potential pathways involve the activation of cyclin-dependent kinase inhibitors (CDKIs), p27 and p21, as mice with a double *p27/p21* knockout exhibit the same phenotype as ERαKO and ArKO models [5,23,28,29].

Sexual dimorphisms in adipose tissue distribution are evident, with men typically having less total body fat but more central and intra-abdominal adipose tissue, while women tend to have more total body fat, primarily in the gluteal/femoral and subcutaneous regions [4]. Metabolic differences and adiposity between males and females are primarily influenced by the location of adipose tissue growth and the bioavailability of estrogens. Subcutaneous fat is generally considered metabolically protective, whereas visceral fat is associated with metabolic dysregulation. Women typically have 10–20% more body fat than men of the same body mass index, further emphasizing estrogen’s influence on fat storage and distribution [8]. Sexual dimorphism in adipose tissue distribution becomes evident during puberty, indicating the role of sex hormones in its development [23].

Lipolysis is regulated in part by adrenergic receptors, with β-adrenergic receptors promoting lipolysis and α2-adrenergic receptors exerting antilipolytic effects. In visceral adipose tissue, adrenaline predominantly stimulates lipolysis due to a higher β-to-α2 receptor ratio, whereas in subcutaneous adipose tissue, the reverse occurs [30]. E2 increases α2-adrenergic receptor expression, which partly explains sex differences in visceral fat distribution and the redistribution of adipose tissue following menopause, characterized by increased visceral fat accumulation [6,30].

A narrative review by Kurylowicz describes evidence explaining the metabolic differences between premenopausal and postmenopausal women in abdominal and femoral subcutaneous adipose tissues, which are potentially linked to variations in ER isotype expression, particularly the Erα-to-ERβ ratio. Premenopausal women exhibited a higher ERα/ERβ ratio compared to postmenopausal women. Moreover, treatment with 17β-E2 in both pre- and postmenopausal subjects increased the ERα/ERβ ratio within white adipose tissue (WAT), suggesting that estrogen-mediated changes in the ER ratio are crucial for enhancing insulin sensitivity in WAT. However, the underlying mechanisms remain unknown. One potential explanation involves DNA methylation, which may contribute to ERα promoter silencing [8].

## 5. Effect of Estrogen on Metabolism from Its Focus Action on Adipose Tissue

Extensive evidence highlights the role of ERs in energy metabolism, primarily derived from animal models and clinical studies. ERs, present even in ancestral invertebrates, initially had a metabolic function rather than a reproductive one [4]. Estrogen’s influence on metabolism is multifaceted, affecting lipolysis, fatty acid oxidation, and lipoprotein lipase (LPL) activity. It promotes fatty acid oxidation and regulates the uptake of free fatty acids, contributing to the overall energy balance and fat distribution. The decline in estrogen levels during menopause leads to increased visceral fat accumulation and heightens risks for metabolic syndromes, including T2D and CVD [4,31,32].

Estrogen plays a key role in lipid metabolism, particularly in suppressing the accumulation of WAT by reducing fatty acid and triglyceride synthesis while enhancing lipolysis. In ovariectomized female mice, E2 decreases adipocyte size by downregulating LPL and other lipogenic enzymes, while simultaneously increasing lipid oxidation in muscle tissue [4].

Postmenopausal women exhibit an increased production of adiponectin, PPARγ, and fatty acid transporters in gluteal adipose tissue, possibly as a compensatory mechanism to preserve insulin sensitivity [33]. Adiponectin, primarily secreted by adipocytes, is found in higher concentrations in women than in men. While its plasma levels generally increase with age and correlate inversely with visceral fat mass, this correlation is weak in postmenopausal women [34]. This may be due to increased free testosterone levels and reduced SHBG concentrations in postmenopausal women, both of which have been linked to lower adiponectin production [35]. Visceral fat accumulation is a key determinant of adiponectin levels, with increased visceral fat leading to decreased adiponectin concentrations [36].

In postmenopausal women, estrogen therapy has been observed to reduce the expression of genes involved in lipogenesis, such as LPL, fatty acid synthase (FAS), and PPARγ. E2 also suppresses lipogenic genes and triglyceride accumulation in WAT and the liver of high-fat diet-fed and leptin-resistant female mice, and the effect was mediated specifically by ERβ agonists rather than ERα agonists. E2 is a major suppressor of fasting LPL activity in adipose tissue and represses LPL gene expression through an estrogen response element on the LPL promoter. Additionally, E2 downregulates the Lipin 1 (LPIN1) gene, which is involved in lipide metabolism and can promote obesity when overexpressed. By regulating LPL activity and LPIN1 expression, E2 helps reduce adipocyte hypertrophy and prevent ectopic lipid accumulation, which are key factors in maintaining metabolic health [4].

Estrogens play a significant role in regulating insulin sensitivity, by enhancing or regulating glucose homeostasis, particularly in adipose tissue, liver, and skeletal muscle [4,37]. Although the chronic administration of E2 has been observed to improve insulin sensitivity in murine models, the effect of E2-mediated insulin-stimulated glucose uptake into muscle remains controversial. Despite its activation of key metabolic pathways, such as protein kinase B (AKT) and adenine monophosphate-activated protein kinase (AMPK), studies have reported heterogeneous results regarding its impact on glucose disposal in skeletal muscle [4].

Research indicates that ERα can influence hepatic insulin sensitivity through mechanisms such as suppressing the ubiquitination-induced degradation of insulin receptor substrate 1 (IRS1) [37].

Physiological and genetic evidence supports the role of E2 and ERs in promoting insulin sensitivity in both sexes when E2 concentrations are within physiological limits. However, supraphysiological levels of E2 or the overstimulation of ERs can induce insulin resistance, potentially due to hyperinsulinemia or reduced GLUT4 expression in muscle [4]. Clinical studies show that premenopausal women have higher insulin sensitivity compared to postmenopausal women and age-matched men. Meta-analyses highlight the protective effect of exogenous estrogens in reducing the risk of insulin resistance and the onset of diabetes. Part of this protective effect can be explained by estrogen’s ability to prevent the accumulation of visceral abdominal fat in female mice, thereby protecting them from developing insulin resistance [23].

In one study, it was shown that female mice lacking ERα globally did not exhibit insulin resistance in skeletal muscle but showed decreased insulin suppression of hepatic glucose production during a euglycemic, hyperinsulinemic clamp, suggesting hepatic insulin resistance due to ERα deficiency. However, Ribas et al. reported only minor alterations in liver insulin sensitivity in conscious, Erα-deficient female mice under similar clamp conditions, indicating that anesthesia might have contributed to the increased hepatic glucose production observed in anesthetized mice [4].

Estrogen plays a role in improving or modulating glucose homeostasis in insulin-sensitive tissues, though the specific actions and mechanisms remain unclear. Further research is needed to understand the molecular pathways involved in these protective effects [37].

Estrogens also regulate adipogenesis through their influence on steroid hormone synthesis. For instance, E2 upregulates the activity of 11-β-hydroxysteroid dehydrogenase type 1 (11β-HSD1), which converts inactive cortisone to active cortisol, a potent up-regulator of adipogenesis in human preadipocytes. The expression of 11β-HSD1 is positively correlated with CYP19A1 and ERβ mRNA levels in subcutaneous adipose tissue (SAT) of both premenopausal and postmenopausal women, irrespective of nutritional status, and is associated with measures of central fat accumulation [23].

Estrogens significantly impact energy regulation; evidence suggests that ERα promotes energy activity by enhancing energy expenditure and reducing caloric intake through the central nervous system. In particular, the ventromedial nucleus (VMN) of the hypothalamus is a crucial area where estrogens exert their effects, modulating thermogenesis and sympathetic activity in response to energy demands. The action of E2 in this region is mediated through pathways such as AMPK, which acts as an energetic sensor. Thus, decreased estrogen levels, such as those experienced during menopause, can lead to a reduction in energy expenditure and an increase in body weight, emphasizing the importance of estrogen in metabolic balance [38].

The metabolic effects of ERβ remain unclear. While ERα gene deletion causes significant metabolic dysfunction in animal models, ERβ gene deletion has minor effects on adiposity or energy balance. This observation led to the hypothesis that ERβ may promote obesity and metabolic disorders. Supporting this, an increase in 17β-E2 concentrations was noted in ERα-null mice, indicating possible enhanced signaling through ERβ. Additionally, ERβ-null mice were initially thought to be protected from obesity after ovariectomy, but later studies showed they were more prone to obesity, yet had protection against insulin resistance. Corresponding human genetic studies have linked five single nucleotide polymorphisms (SNPs) in ERβ to obesity in both males and females [8].

Recent research indicates that ERβ could have a protective metabolic role by regulating WAT mitochondrial activity. ERβ-specific ligands have been shown to increase energy expenditure and WAT mitochondrial activity in rodents, independent of circulating estrogens. These findings suggest that both ERα and ERβ are essential for optimal metabolic function and flexibility. However, further studies are required to fully understand the roles of ER isotype activation, expression profiling, receptor–DNA interactions, and estrogen’s non-genomic activities [4,8].

## 6. Premature Ovarian Insufficiency

POI encompasses a spectrum of disorders characterized by impaired ovarian function due to follicular dysfunction or depletion. Although the precise pathophysiological mechanisms remain unclear, POI has been historically referred to by various terms, including premature ovarian failure, primary ovarian insufficiency, decreased ovarian reserve, and premature menopause. To standardize terminology and enhance international communication, the European Society of Human Reproduction and Embryology (ESHRE) adopted the term “premature ovarian insufficiency” in 2013 [39].

POI is clinically defined by amenorrhea lasting at least four months, accompanied by elevated follicle-stimulating hormone (FSH) levels (>25 IU/L) and decreased E2 concentrations. Diagnosis requires confirmation through repeated hormonal assessments at least one month apart in women under 40 years of age. Secondary causes of amenorrhea, including pregnancy, thyroid dysfunction, and hyperprolactinemia, must be excluded [40].

Etiologies of POI are diverse, encompassing genetic and autoimmune disorders, infections, chromosomal abnormalities (e.g., Turner syndrome), Fragile X syndrome, and iatrogenic factors, such as chemotherapy, radiation, and ovarian surgery. However, up to 90% of cases remain idiopathic. Notably, mutations in genes encoding LH and FSH receptors have been implicated in disrupted gonadal function [41].

## 7. Metabolic Alterations in Premature Ovarian Insufficiency

There is a complex interplay between ovarian function, adipose tissue, and metabolic homeostasis. Women with POI exhibit a significantly increased risk of CVD and a reduced life expectancy, driven by endothelial dysfunction, dyslipidemia, insulin resistance, and metabolic syndrome [42,43,44].

(a) Insulin Sensitivity and Resistance Mechanism in POI

Both early menopause (EM) and POI have been associated with an elevated risk of T2D. A meta-analysis by Anagnostis et al. (2019) reported a higher likelihood of T2D in women with EM and POI compared to those undergoing menopause at the typical age of 45–55 years (OR for EM: 1.15, 95% CI: 1.04–1.26; OR for POI: 1.50, 95% CI: 1.03–2.19) [45]. Overall, women with POI face a 53% increased risk of developing T2D [43].

The mechanisms underlying this association are multifaceted and likely involve estrogen deficiency, which impacts pancreatic β-cell function, insulin biosynthesis, and glucose homeostasis. E2, acting via ERα activation and extracellular signal-regulated kinase (ERK1/2) phosphorylation, enhances insulin secretion and β-cell survival. Reduced estrogen levels in POI contribute to glucose intolerance, central adiposity, and increased insulin resistance [45].

Studies have demonstrated elevated fasting insulin levels in women with POI compared to controls, particularly during oral glucose tolerance tests (OGTT) at 0, 60, and 120 min post-ingestion [46,47]. However, a study of 98 women with idiopathic POI and normal karyotypes found no significant differences in insulin resistance indices (quantitative insulin sensitivity check index (QUICKI), homeostatic model assessment (HOMA), Matsuda, McAuley index (McA), and fasting glucose-to-insulin ratio (FGIR)) between POI patients and controls. Despite this, QUICKI exhibited the highest sensitivity (*p* < 0.05) in detecting insulin resistance, identifying a prevalence of 28.57% in POI compared to 23.07% in controls [47].

Adipokines, such as adiponectin, and pro-inflammatory markers, including tumoral necrosis factor alfa (TNFα) and interleukin 6 (IL-6), have been implicated in insulin resistance and systemic inflammation in POI [48].

(b) Adipose Tissue and Lipid Metabolism in POI

FSH and its receptors play a crucial role in metabolic homeostasis, with potential implications for metabolic disorders such as obesity, T2D, and non-alcoholic fatty liver disease. Estrogen deficiency in POI leads to metabolic disturbances comparable to those observed in postmenopausal women [46].

The metabolic profile of POI has been partially extrapolated from studies on menopause [49]. Estrogen decline, alongside a less-pronounced reduction in androgens, results in an increased androgen-to-estrogen ratio, continued androgen synthesis by adrenal and ovarian thecal cells, and decreased SHBG levels. These hormonal alterations contribute to increased total and visceral adiposity, decreased lean mass, and ectopic fat deposition in the liver and skeletal muscle. Central obesity, characterized by chronic inflammation and oxidative stress, further exacerbates insulin resistance through increased free fatty acid release from visceral adipocytes [50,51].

E2 exerts cardioprotective effects by modulating lipid metabolism, reducing total cholesterol (TC) and LDL levels, and enhancing LDL receptor activity. It also inhibits hepatic lipase, preserves HDL levels, and facilitates LDL clearance. In POI, E2 deficiency leads to oxidative stress, endothelial dysfunction, and activation of the renin–angiotensin system, promoting atherosclerosis and vascular aging [48]. Epidemiological studies report an increased hazard ratio (HR) of 1.61 for CVD and an elevated risk of stroke in women with POI. Notably, women with natural POI appear to have a higher genetic predisposition to vascular complications than those with surgically induced POI [43].

A meta-analysis by Wang et al. (2022) identified significantly elevated TC, LDL, and triglyceride levels in POI patients compared to healthy controls [52]. Insulin resistance contributes to hypertriglyceridemia by reducing LPL activity, impairing triglyceride metabolism, and promoting lipid accumulation key risk factors for CVD [52]. Women with POI have been reported to exhibit lower leptin concentrations [53], a finding corroborated by Benetti-Pinto et al. [54].

(c) Steroidogenesis and Lipid Dysregulation in POI

Steroidogenesis may play a role in the metabolic abnormalities observed in women with POI. This process involves the conversion of cholesterol into estrogen within the ovaries, with oocytes depending on serum lipids, particularly cholesteryl esters, for granulosa cell steroidogenesis. Impaired estrogen production in POI may lead to increased circulating lipid levels. Notably, women with POI showed significantly higher HDL levels than controls, likely because HDL serves as the primary cholesterol transporter to granulosa cells during steroidogenesis. The findings suggest that reduced estrogen synthesis in POI may contribute to lipid accumulation, though unadjusted confounders, such as age and body mass index (BMI), may also influence lipid levels [46].

## 8. Cardiovascular Disease in POI

Although CVD has been traditionally viewed as a disease of postmenopausal women—attributed to the decline in protective estrogen levels—emerging evidence suggests that EM and diminished ovarian reserve are also associated with an increased cardiovascular risk. These conditions provide an opportunity for the early identification of women at high risk for CVD, allowing for targeted preventive interventions [55,56,57,58,59]. In a cohort of well-characterized women with POI, Christ et al. demonstrated that estrogen deprivation is associated with an increased risk of CVD, whereas sustained estrogen exposure is linked to a reduced risk [60].

Under physiological conditions, the transition to menopause and post-menopause is characterized by a decline or absence of estrogen, leading to lipoprotein alterations associated with increased cardiovascular risk. Total cholesterol, LDL, and apolipoprotein B levels rise approximately one year before the final menstrual period, while HDL levels tend to decrease during this transition. These lipid alterations contribute to endothelial dysfunction and promote atherosclerosis [61,62].

The relationship between metabolic dysfunction and POI is complex. A cross-sectional study conducted by Chu et al. analyzed women aged 25–55 years with regular menstrual cycles, who were previously healthy and free from CVD or hyperlipidemia. The study found that premenopausal women with FSH concentrations > 7 IU/L had significantly higher total cholesterol (4.93 mmol/L vs. 4.38 mmol/L, *p* = 0.009) and LDL levels (3.05 mmol/L vs. 2.52 mmol/L, *p* = 0.019) compared to those with FSH < 7 IU/L. This correlation was independent of age (*p* = 0.03) [63].

In patients with POI, findings on serum lipid profiles remain inconsistent. Several studies have analyzed lipid levels in POI patients, yielding contradictory results [64]. A 2021 meta-analysis comparing serum triglycerides, HDL, and LDL levels between POI patients and healthy controls reported significantly higher total cholesterol, triglyceride, and LDL levels in POI patients. However, HDL levels did not differ significantly between groups [52]. This dyslipidemic profile is associated with an increased cardiovascular risk, potentially contributing to an up to 80% higher mortality rate from ischemic heart disease in women with POI compared to those who experience menopause at the typical age of 49–55 years [64,65].

In a case-control study by Ates et al., the metabolic profile and abdominal fat distribution of karyotypically normal women with POI were compared to those without POI. Women with POI exhibited higher total cholesterol levels (191.66 ± 33.27 vs. 174.86 ± 37.22 mg/dL, *p* = 0.013) and HDL (60.88 ± 16.41 vs. 53.90 ± 11.96, *p* = 0.011), as well as a higher prevalence of metabolic syndrome (14.3% vs. 3.4%, *p* = 0.049). However, no significant differences were observed in insulin sensitivity or adipose tissue distribution [66]. Similar findings were reported in studies by Podfigurna et al. and Huang et al. [44,67]. In contrast, a cohort study by Corrigan et al. found that POI was not associated with increased central or total adiposity but was linked to lower insulin sensitivity. However, this study included patients with Monosomy 45X [68].

These and additional studies were incorporated into a meta-analysis by Cai et al., which concluded that patients with POI had significantly higher waist circumference, total cholesterol, LDL, HDL, triglycerides, and fasting glucose, but no significant differences in systolic or diastolic blood pressure [46]. As mentioned above, a meta-analysis by Wang et al. corroborated these findings, except for HDL, where no significant difference was observed [52]. A more recent study by Jin et al. found no significant difference in the prevalence of metabolic syndrome among women with POI. However, significant differences were noted in the prevalence of hypertriglyceridemia and impaired fasting glucose, while other metabolic syndrome components, including abdominal circumference, showed no significant variation [69]. Regarding adipokines, women with surgically induced POI, administration of E2 (1 mg/day), or tibolone (2.5 mg/day) did not significantly alter leptin concentrations at 5 days or 3 months of treatment [70].

(a) Hormone replacement therapy effects in POI

There is limited information on the long-term metabolic effects of hormone replacement therapy (HRT) in women with POI, as most studies have primarily focused on bone health [71,72]. However, the potential cardiovascular benefits of HRT can be extrapolated from data on postmenopausal women, given that prospective studies suggest that women with POI have a CVD risk comparable to that of postmenopausal women [73]. In this context, HRT plays a crucial role in preventing diseases associated with early estrogen deficiency in POI patients [74].

While the effects of HRT on the lipid profile in POI remain unclear, data from women with natural menopause suggest that oral estrogen therapy reduces total cholesterol and LDL levels while increasing HDL concentrations [64,75,76]. Further research is needed to elucidate the impact of HRT on lipid metabolism in women with POI.

Furthermore, POI has been associated with endothelial dysfunction, which can be ameliorated with HRT [77]. Endothelial dysfunction is an early marker of preclinical atherosclerosis, and in some cases, pharmacological interventions may reverse this process. In patients with POI, endothelial function measured as flow-mediated dilation of the brachial artery is reduced. Additionally, these patients exhibit increased carotid intima-media thickness and altered left ventricular diastolic function. Notably, these vascular abnormalities have been shown to improve within six months of HRT initiation, underscoring the importance of early diagnosis and intervention to mitigate cardiovascular risk in this population [64,78,79,80].

Systemic arterial hypertension is one of the most prevalent chronic conditions in women, regardless of POI status. A study reported similar prevalence rates of hypertension in both POI patients (12%) and controls (19%), with no statistically significant differences between groups. In women with previous hypertension who undergo menopause at a physiological age, initiating HRT within the first 10 years of amenorrhea or before the age of 60 has been associated with improved blood pressure control. However, the effects of HRT on blood pressure regulation in women with POI remain unclear and warrant further investigation [64,75,81]. The impact of POI on myocardial function remains unclear. However, some studies indicate that the myocardial performance index in patients with POI is significantly impaired compared to healthy controls (0.87 ± 0.27 vs. 0.5 ± 0.2, *p* < 0.001), while no significant difference has been observed in the pulse wave velocity index [82].

(b) Mortality in woman with POI

Mortality is higher in this population. In a cohort study of 1159 Chilean women with a follow-up of 29.2 years (28.6–29.7), Blümed et al. reported that women with POI had a higher overall mortality rate compared to those without ovarian insufficiency (34.7% vs. 19.3%, *p* < 0.001), as well as a great risk of CVD-related death (12.0% vs. 5.1%; OR 2.55, CI 95%: 1.21–5.39) [83]. Similarly, a 2016 meta-analysis by Roeters van Lennep et al., which included 10 observational studies and a total of 190,588 women, found that POI was associated with an increased risk of death due to ischemic heart disease (HR 1.69, 95% IC: 1.29–2.21, *p* = 0.0001) [84].

These findings are particularly relevant, as more than half of women with POI have been reported to either never initiate HRT, begin treatment years after diagnosis, or discontinue it prematurely. Moreover, for each year a woman with POI remains without E2 exposure, her risk of experiencing a hard or general CVD event increases by 0.18–2.0%. Conversely, for every year of E2 exposure, this risk decreases by 0.15–0.16% [60].

## 9. Summary and Conclusions

Estrogens play a crucial role in regulating adipogenesis and body fat distribution. They influence the differentiation and proliferation and preadipocytes through various cellular and molecular mechanisms. In particular, ERα is essential for determining the fate of APCs. The absence of ERα leads to lipodystrophy and a shift toward myofibrotic lineages. Additionally, estrogens, synthesized in adipocytes from androgenic precursors, are directly linked to total body adiposity. The elimination of ERα in animal models is associated with a significant increase in adiposity, especially in visceral fat depots, highlighting its key role in fat storage regulation.

Studies have shown that mice with a global ERα knockout exhibit a 50–180% increase in adipocyte number, along with insulin resistance, glucose intolerance, and hepatic steatosis. Key mechanisms regulating adipose metabolism include the inhibition of PPARγ and the activation of CDKIs such as p27 and p21. Regarding fat distribution, there are marked sex differences: men tend to have less total fat but greater visceral fat accumulation, whereas women predominantly store fat in the subcutaneous region, particularly in the gluteal and femoral areas. This distribution changes after menopause due to the decline in estrogen levels, promoting visceral fat storage and a less favorable metabolic profile.

Estrogens also play a significant role in lipid metabolism by reducing fatty acid and triglyceride synthesis while promoting lipid oxidation. In animal models of induced menopause, E2 reduces adipocyte size by suppressing LPL and other lipogenic enzymes while increasing lipid oxidation in muscle tissue. In postmenopausal women, adiponectin production and PPARγ expression in gluteal adipose tissue increase, potentially acting as a compensatory mechanism to maintain insulin sensitivity. However, the correlation between adiponectin and visceral fat is weak in these women, possibly due to higher levels of free testosterone and reduced SHBG.

In POI, a significant metabolic impact is observed, characterized by insulin resistance, dyslipidemia, and a increased cardiovascular risk. Women with POI have a 53% higher risk of developing T2D compared to those experiencing menopause at the typical age. Estrogen deficiency affects pancreatic β-cell function, insulin biosynthesis, and glucose homeostasis. Additionally, central obesity and ectopic fat accumulation in the liver and skeletal muscle contribute to insulin resistance, exacerbated by an increased androgen-to-estrogen ratio.

Estrogen also regulates insulin sensitivity through their interaction with ERα in the liver and adipose tissue. Their deficiency in animal models induces hepatic insulin resistance, while in humans, estrogen therapy has shown protective effects by reducing visceral fat accumulation and improving insulin sensitivity. However, supraphysiological estrogen levels can induce insulin resistance through hyperinsulinemia or reduced GLUT4 expression in muscle tissue.

**Figure 2:**
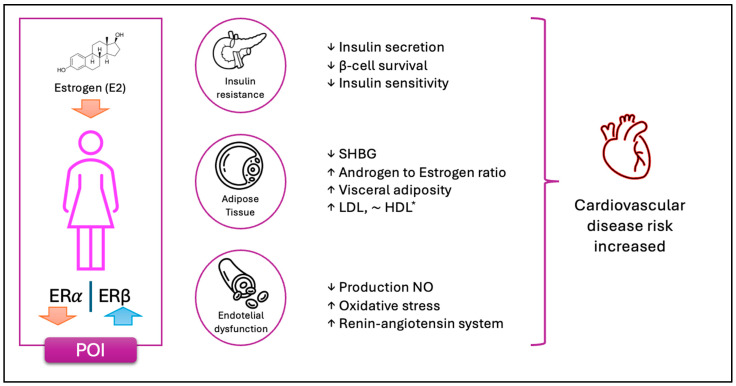
The schematic representation illustrates the key physiological changes observed in women with POI. The depletion of ovarian follicles leads to a loss of granulosa cells, resulting in a marked reduction in E2 production. This hormonal decline disrupts the balance between ERα and ERβ expression, contributing to various metabolic alterations. Notably, these changes predispose individuals to insulin resistance and impairments in carbohydrate metabolism. The diminished estrogenic signaling compromises its insulin-sensitizing and anti-apoptotic effects on pancreatic beta cells and adipose tissue. Additionally, dysregulated steroidogenesis leads to increased androgen production and elevated levels of free androgens, promoting visceral fat accumulation. Further metabolic consequences include reduced clearance of LDL cholesterol. Moreover, the absence of estrogen-mediated endothelial activation results in increased oxidative stress due to diminished nitric oxide (NO) production. This hormonal deficiency also enhances the activation of the renin–angiotensin system, driven by both elevated angiotensinogen synthesis in adipose tissue and impaired vascular vasodilation. Collectively, these factors contribute to an increased cardiovascular risk in women with POI. For *HDL, the evidence is contradictory. While HDL concentrations appear to be slightly higher compared to controls, some studies suggest both an increase and a decrease in HDL levels following a reduction in E2 levels.

In conclusion, estrogens play a key role in regulating adipogenesis, fat distribution and lipid and glucose metabolism. Their decline in menopause or POI leads to metabolic changes that increase the risk of central obesity, insulin resistance, and cardiovascular disease. Understanding these mechanisms could contribute to the development of more effective therapeutic strategies to prevent and treat metabolic disorders associated with estrogen deficiency (see Figure 2).

## Data Availability

Not applicable.

## Abbreviations

The following abbreviations are used in this manuscript:

TC	Total cholesterol
LDL	Low-density lipoprotein
HDL	High-density lipoprotein
LPL	Lipoprotein lipase
E1	Estrone
E2	Estradiol
E3	Estriol
E4	Estetrol
DHEA	Dehydroepiandrosterone
DHEA-S	Dehydroepiandrosterone sulfate
A4	Androstenedione
LH	Luteinizing hormone
FSH	Follicle-stimulating hormone
SHBG	Sex hormone-binding globulin
DRD	Dimerization and regulatory domain
GLUT4	Glucose transporter type 4
GPER	G-protein coupled estrogen receptor
ERα (ESR1 o ER1)	Estrogen receptor alpha (type 1)
ERβ (ESR2 o ER2)	Estrogen receptor beta (type 2)
ERs	Estrogen receptors
EREs	Estrogen response elements
SRC	Steroid receptor coactivator
AKT	Protein kinase B
AMPK	Adenine monophosphate-activated protein kinase
ERαKO	ER alpha knockout
ArKO	Aromatase knockout
CYP	Cytochrome P450
UDP	Uridine diphosphate
COMT	Catechol-O-methyltransferase
CYP19A1	Cytochrome P450 family 19 subfamily A member 1 (Aromatase gene)
11β-HSD1	11β-hydroxysteroid dehydrogenase type 1
SNP	Single nucleotide polymorphism
cAMP	Cyclic adenine monophosphate
APC	Adipose progenitor cells
CREB	cAMP response element binding protein
CBP	CREB-binding protein
WAT	White adipose tissue
SAT	Subcutaneous adipose tissue
LPIN1	Lipin 1
FAS	Fatty acid synthase
VMN	Ventromedial nucleus
DNA	Deoxyribonucleic acid
ESHRE	European Society of Human Reproduction and Embryology
EM	Early menopause
ERK1/2	Extracellular signal-regulated kinase
QUICKI	Quantitative insulin sensitivity check index
HOMA	Homeostatic model assessment
McA	McAuley index
FGIR	Fasting Glucose to Insulin Ratio
HR	Hazard ratio
HRT	Hormone replacement therapy
ISR-1	Insulin receptor substrate 1
PPARγ	Peroxisome proliferator-activated receptor gamma
POI	Premature ovarian insufficiency
CVD	Cardiovascular disease
T2D	Type 2 diabetes
ADKIs	Cyclin-dependent kinase inhibitors

## References

[1] Eyster K.M. (2016). The Estrogen Receptors: An Overview from Different Perspectives. Methods Mol. Biol..

[2] Vrtacnik P., Ostanek B., Mencej-Bedrac S., Marc J. (2014). The many faces of estrogen signaling. Biochem. Med..

[3] Rettberg J.R., Yao J., Brinton R.D. (2014). Estrogen: A master regulator of bioenergetic systems in the brain and body. Front. Neuroendocrinol..

[4] Steiner B.M., Berry D.C. (2022). The Regulation of Adipose Tissue Health by Estrogens. Front. Endocrinol..

[5] Faulds M.H., Zhao C., Dahlman-Wright K., Gustafsson J.A. (2012). The diversity of sex steroid action: Regulation of metabolism by estrogen signaling. J. Endocrinol..

[6] Gupte A.A., Pownall H.J., Hamilton D.J. (2015). Estrogen: An emerging regulator of insulin action and mitochondrial function. J. Diabetes Res..

[7] Khalil R.A. (2010). Potential approaches to enhance the effects of estrogen on senescent blood vessels and postmenopausal cardiovascular disease. Cardiovasc. Hematol. Agents Med. Chem..

[8] Kurylowicz A. (2023). Estrogens in Adipose Tissue Physiology and Obesity-Related Dysfunction. Biomedicines.

[9] Alemany M. (2021). Estrogens and the regulation of glucose metabolism. World J. Diabetes.

[10] Frederiksen H., Johannsen T.H., Andersen S.E., Albrethsen J., Landersoe S.K., Petersen J.H., Andersen A.N., Vestergaard E.T., Schorring M.E., Linneberg A. (2020). Sex-specific Estrogen Levels and Reference Intervals from Infancy to Late Adulthood Determined by LC-MS/MS. J. Clin. Endocrinol. Metab..

[11] Falah N., Torday J., Quinney S., Haas D. (2015). Estriol review: Clinical applications and potential biomedical importance. Clin. Res. Trials.

[12] Buscato M., Davezac M., Zahreddine R., Adlanmerini M., Metivier R., Fillet M., Cobraiville G., Moro C., Foidart J.M., Lenfant F. (2021). Estetrol prevents Western diet-induced obesity and atheroma independently of hepatic estrogen receptor α. Am. J. Physiol. Endocrinol. Metab..

[13] Levin E.R. (2009). Plasma membrane estrogen receptors. Trends Endocrinol. Metab..

[14] Hewitt S.C., Korach K.S. (2018). Estrogen Receptors: New Directions in the New Millennium. Endocr. Rev..

[15] Couse J.F., Curtis S.W., Washburn T.F., Lindzey J., Golding T.S., Lubahn D.B., Smithies O., Korach K.S. (1995). Analysis of transcription and estrogen insensitivity in the female mouse after targeted disruption of the estrogen receptor gene. Mol. Endocrinol..

[16] Hamilton K.J., Arao Y., Korach K.S. (2014). Estrogen hormone physiology: Reproductive findings from estrogen receptor mutant mice. Reprod. Biol..

[17] Bryzgalova G., Gao H., Ahren B., Zierath J.R., Galuska D., Steiler T.L., Dahlman-Wright K., Nilsson S., Gustafsson J.A., Efendic S. (2006). Evidence that oestrogen receptor-α plays an important role in the regulation of glucose homeostasis in mice: Insulin sensitivity in the liver. Diabetologia.

[18] Hodgin J.B., Krege J.H., Reddick R.L., Korach K.S., Smithies O., Maeda N. (2001). Estrogen receptor α is a major mediator of 17β-estradiol’s atheroprotective effects on lesion size in Apoe^−/−^ mice. J. Clin. Investig..

[19] Svenson J.L., EuDaly J., Ruiz P., Korach K.S., Gilkeson G.S. (2008). Impact of estrogen receptor deficiency on disease expression in the NZM2410 lupus prone mouse. Clin. Immunol..

[20] Le May C., Chu K., Hu M., Ortega C.S., Simpson E.R., Korach K.S., Tsai M.J., Mauvais-Jarvis F. (2006). Estrogens protect pancreatic β-cells from apoptosis and prevent insulin-deficient diabetes mellitus in mice. Proc. Natl. Acad. Sci. USA.

[21] Sims N.A., Dupont S., Krust A., Clement-Lacroix P., Minet D., Resche-Rigon M., Gaillard-Kelly M., Baron R. (2002). Deletion of estrogen receptors reveals a regulatory role for estrogen receptors-β in bone remodeling in females but not in males. Bone.

[22] Prossnitz E.R., Hathaway H.J. (2015). What have we learned about GPER function in physiology and disease from knockout mice?. J. Steroid Biochem. Mol. Biol..

[23] Bjune J.I., Stromland P.P., Jersin R.A., Mellgren G., Dankel S.N. (2022). Metabolic and Epigenetic Regulation by Estrogen in Adipocytes. Front. Endocrinol..

[24] Saavedra-Peña R.D.M., Taylor N., Flannery C., Rodeheffer M.S. (2023). Estradiol cycling drives female obesogenic adipocyte hyperplasia. Cell Rep..

[25] Fuentes N., Silveyra P. (2019). Estrogen receptor signaling mechanisms. Adv. Protein Chem. Struct. Biol..

[26] Heine P.A., Taylor J.A., Iwamoto G.A., Lubahn D.B., Cooke P.S. (2000). Increased adipose tissue in male and female estrogen receptor-α knockout mice. Proc. Natl. Acad. Sci. USA.

[27] Jones M.E., Thorburn A.W., Britt K.L., Hewitt K.N., Wreford N.G., Proietto J., Oz O.K., Leury B.J., Robertson K.M., Yao S. (2000). Aromatase-deficient (ArKO) mice have a phenotype of increased adiposity. Proc. Natl. Acad. Sci. USA.

[28] Jeong S., Yoon M. (2011). 17β-Estradiol inhibition of PPARγ-induced adipogenesis and adipocyte-specific gene expression. Acta Pharmacol. Sin..

[29] Naaz A., Holsberger D.R., Iwamoto G.A., Nelson A., Kiyokawa H., Cooke P.S. (2004). Loss of cyclin-dependent kinase inhibitors produces adipocyte hyperplasia and obesity. FASEB J..

[30] Pedersen S.B., Kristensen K., Hermann P.A., Katzenellenbogen J.A., Richelsen B. (2004). Estrogen controls lipolysis by up-regulating α2A-adrenergic receptors directly in human adipose tissue through the estrogen receptor α. Implications for the female fat distribution. J. Clin. Endocrinol. Metab..

[31] Chait A., den Hartigh L.J. (2020). Adipose Tissue Distribution, Inflammation and Its Metabolic Consequences, Including Diabetes and Cardiovascular Disease. Front. Cardiovasc. Med..

[32] Pallottini V., Bulzomi P., Galluzzo P., Martini C., Marino M. (2008). Estrogen regulation of adipose tissue functions: Involvement of estrogen receptor isoforms. Infect. Disord.-Drug Targets.

[33] Misso M.L., Jang C., Adams J., Tran J., Murata Y., Bell R., Boon W.C., Simpson E.R., Davis S.R. (2005). Differential expression of factors involved in fat metabolism with age and the menopause transition. Maturitas.

[34] Koh S.J., Hyun Y.J., Choi S.Y., Chae J.S., Kim J.Y., Park S., Ahn C.M., Jang Y., Lee J.H. (2008). Influence of age and visceral fat area on plasma adiponectin concentrations in women with normal glucose tolerance. Clin. Chim. Acta.

[35] Sieminska L., Cichon-Lenart A., Kajdaniuk D., Kos-Kudla B., Marek B., Lenart J., Nowak M. (2006). Sex hormones and adipocytokines in postmenopausal women. Polski Merkur. Lek..

[36] Ritland L.M., Alekel D.L., Matvienko O.A., Hanson K.B., Stewart J.W., Hanson L.N., Reddy M.B., Van Loan M.D., Genschel U. (2008). Centrally located body fat is related to appetitive hormones in healthy postmenopausal women. Eur. J. Endocrinol..

[37] De Paoli M., Zakharia A., Werstuck G.H. (2021). The Role of Estrogen in Insulin Resistance: A Review of Clinical and Preclinical Data. Am. J. Pathol..

[38] Lizcano F. (2022). Roles of estrogens, estrogen-like compounds, and endocrine disruptors in adipocytes. Front. Endocrinol..

[39] Chon S.J., Umair Z., Yoon M.S. (2021). Premature Ovarian Insufficiency: Past, Present, and Future. Front. Cell Dev. Biol..

[40] Wesevich V., Kellen A.N., Pal L. (2020). Recent advances in understanding primary ovarian insufficiency. F1000Research.

[41] Rudnicka E., Kruszewska J., Klicka K., Kowalczyk J., Grymowicz M., Skorska J., Pieta W., Smolarczyk R. (2018). Premature ovarian insufficiency—Aetiopathology, epidemiology, and diagnostic evaluation. Prz. Menopauzalny.

[42] Ishizuka B. (2021). Current Understanding of the Etiology, Symptomatology, and Treatment Options in Premature Ovarian Insufficiency (POI). Front. Endocrinol..

[43] Stevenson J.C., Collins P., Hamoda H., Lambrinoudaki I., Maas A.H.E.M., Maclaran K., Panay N. (2021). Cardiometabolic health in premature ovarian insufficiency. Climacteric.

[44] Podfigurna A., Stellmach A., Szeliga A., Czyzyk A., Meczekalski B. (2018). Metabolic Profile of Patients with Premature Ovarian Insufficiency. J. Clin. Med..

[45] Anagnostis P., Christou K., Artzouchaltzi A.M., Gkekas N.K., Kosmidou N., Siolos P., Paschou S.A., Potoupnis M., Kenanidis E., Tsiridis E. (2019). Early menopause and premature ovarian insufficiency are associated with increased risk of type 2 diabetes: A systematic review and meta-analysis. Eur. J. Endocrinol..

[46] Cai W.Y., Luo X., Wu W., Song J., Xie N.N., Duan C., Wu X.K., Xu J. (2022). Metabolic differences in women with premature ovarian insufficiency: A systematic review and meta-analysis. J. Ovarian Res..

[47] Kunicki M., Rudnicka E., Skórska J., Calik-Ksepka A.I., Smolarczyk R. (2018). Insulin resistance indexes in women with premature ovarian insufficiency—A pilot study. Ginekol. Polska.

[48] Huang L., Wang H., Shi M., Kong W., Jiang M. (2022). Lipid Profile in Patients With Primary Ovarian Insufficiency: A Systematic Review and Meta-Analysis. Front. Endocrinol..

[49] Wellons M. (2011). Cardiovascular disease and primary ovarian insufficiency. Semin. Reprod. Med..

[50] Lambrinoudaki I., Armeni E. (2024). Understanding of and clinical approach to cardiometabolic transition at the menopause. Climacteric.

[51] Daan N.M.P., Muka T., Koster M.P.H., van Lennep J.E.R., Lambalk C.B., Laven J.S.E., Fauser C.G.K.M., Meun C., de Rijke Y.B., Boersma E. (2016). Cardiovascular Risk in Women With Premature Ovarian Insufficiency Compared to Premenopausal Women at Middle Age. J. Clin. Endocrinol. Metab..

[52] Wang Z., Fang L., Wu Z., Li Y., Jia Q., Cheng J.C., Sun Y.P. (2022). A meta-analysis of serum lipid profiles in premature ovarian insufficiency. Reprod. Biomed. Online.

[53] Sarac F., Oztekin K., Celebi G. (2011). Early menopause association with employment, smoking, divorced marital status and low leptin levels. Gynecol. Endocrinol..

[54] Benetti-Pinto C.L., Castro N., da Rocha Grassiotto O., Garmes H.M. (2010). Leptin and adiponectin blood levels in women with premature ovarian failure and age- and weight-matched women with normal menstrual cycles. Menopause.

[55] Quinn M.M., Cedars M.I. (2018). Cardiovascular health and ovarian aging. Fertil. Steril..

[56] Schipper I., Louwers Y.V. (2020). Premature and Early Menopause in Relation to Cardiovascular Disease. Semin. Reprod. Med..

[57] Gunning M.N., Meun C., van Rijn B.B., Daan N.M.P., van Lennep J.E.R., Appelman Y., Boersma E., Hofstra L., Fauser C.G.K.M., Rueda-Ochoa O.L. (2020). The cardiovascular risk profile of middle age women previously diagnosed with premature ovarian insufficiency: A case-control study. PLoS ONE.

[58] Gunning M.N., Meun C., van Rijn B.B., Maas A.H.E.M., Benschop L., Franx A., Boersma E., Budde R.P.J., Appelman Y., Lambalk C.B. (2019). Coronary artery calcification in middle-aged women with premature ovarian insufficiency. Clin. Endocrinol..

[59] Podfigurna A., Meczekalski B. (2018). Cardiovascular health in patients with premature ovarian insufficiency. Management of long-term consequences. Prz. Menopauzalny.

[60] Christ J.P., Gunning M.N., Palla G., Eijkemans M.J.C., Lambalk C.B., Laven J.S.E., Fauser B. (2018). Estrogen deprivation and cardiovascular disease risk in primary ovarian insufficiency. Fertil. Steril..

[61] Wu B., Fan B., Qu Y., Li C., Chen J., Liu Y., Wang J., Zhang T., Chen Y. (2023). Trajectories of Blood Lipids Profile in Midlife Women: Does Menopause Matter?. J. Am. Heart Assoc..

[62] Lou Z., Huang Y., Lan Y., Li C., Chu K., Chen P., Xu W., Ma L., Zhou J. (2023). Relationship between years since menopause and lipid variation in postmenopausal women: A cross-sectional study. Medicine.

[63] Chu M.C., Rath K.M., Huie J., Taylor H.S. (2003). Elevated basal FSH in normal cycling women is associated with unfavourable lipid levels and increased cardiovascular risk. Hum. Reprod..

[64] Podfigurna-Stopa A., Czyzyk A., Grymowicz M., Smolarczyk R., Katulski K., Czajkowski K., Meczekalski B. (2016). Premature ovarian insufficiency: The context of long-term effects. J. Endocrinol. Investig..

[65] Jacobsen B.K., Knutsen S.F., Fraser G.E. (1999). Age at natural menopause and total mortality and mortality from ischemic heart disease: The Adventist Health Study. J. Clin. Epidemiol..

[66] Ates S., Yesil G., Sevket O., Molla T., Yildiz S. (2014). Comparison of metabolic profile and abdominal fat distribution between karyotypically normal women with premature ovarian insufficiency and age matched controls. Maturitas.

[67] Huang Y., Lv Y., Qi T., Luo Z., Meng X., Ying Q., Li D., Li C., Lan Y., Chu K. (2021). Metabolic profile of women with premature ovarian insufficiency compared with that of age-matched healthy controls. Maturitas.

[68] Corrigan E.C., Nelson L.M., Bakalov V.K., Yanovski J.A., Vanderhoof V.H., Yanoff L.B., Bondy C.A. (2006). Effects of ovarian failure and X-chromosome deletion on body composition and insulin sensitivity in young women. Menopause.

[69] Jin J., Ruan X., Hua L., Mueck A.O. (2023). Prevalence of metabolic syndrome and its components in Chinese women with premature ovarian insufficiency. Gynecol. Endocrinol..

[70] Petzel M., Stejskal D., Jedelsky L., Kadalova L., Safarcik K. (2008). The influence of estradiole and tibolone administration on leptin levels in women with surgically induced menopause. Biomed. Pap. Med. Fac. Palacky Univ. Olomouc.

[71] Burgos N., Cintron D., Latortue-Albino P., Serrano V., Rodriguez Gutierrez R., Faubion S., Spencer-Bonilla G., Erwin P.J., Murad M.H. (2017). Estrogen-based hormone therapy in women with primary ovarian insufficiency: A systematic review. Endocrine.

[72] Crofton P.M., Evans N., Bath L.E., Warner P., Whitehead T.J., Critchley H.O., Kelnar C.J., Wallace W.H. (2010). Physiological versus standard sex steroid replacement in young women with premature ovarian failure: Effects on bone mass acquisition and turnover. Clin. Endocrinol..

[73] Lersten I., Clain E., Santoro N. (2020). Use of Hormone Therapy in Women with Early Menopause and Premature Ovarian Insufficiency. Semin. Reprod. Med..

[74] Faubion S.S., Kuhle C.L., Shuster L.T., Rocca W.A. (2015). Long-term health consequences of premature or early menopause and considerations for management. Climacteric.

[75] Sullivan S.D., Sarrel P.M., Nelson L.M. (2016). Hormone replacement therapy in young women with primary ovarian insufficiency and early menopause. Fertil. Steril..

[76] Rezende G.P., Dassie T., Gomes D.A.Y., Benetti-Pinto C.L. (2023). Cardiovascular Risk Factors in Premature Ovarian Insufficiency using Hormonal Therapy. Rev. Bras. Ginecol. Obstet..

[77] Kalantaridou S.N., Naka K.K., Papanikolaou E., Kazakos N., Kravariti M., Calis K.A., Paraskevaidis E.A., Sideris D.A., Tsatsoulis A., Chrousos G.P. (2004). Impaired endothelial function in young women with premature ovarian failure: Normalization with hormone therapy. J. Clin. Endocrinol. Metab..

[78] Kalantaridou S.N., Naka K.K., Bechlioulis A., Makrigiannakis A., Michalis L., Chrousos G.P. (2006). Premature ovarian failure, endothelial dysfunction and estrogen-progestogen replacement. Trends Endocrinol. Metab..

[79] Goldmeier S., De Angelis K., Casali K.R., Vilodre C., Consolim-Colombo F., Klein A.B., Plentz R., Spritzer P., Irigoyen M.C. (2013). Cardiovascular autonomic dysfunction in primary ovarian insufficiency: Clinical and experimental evidence. Am. J. Transl. Res..

[80] Yorgun H., Gurses K.M., Canpolat U., Yapici Z., Bozdag G., Kaya E.B., Aytemir K., Oto A., Kabakci G., Tokgozoglu L. (2012). Evaluation of cardiac autonomic function by various indices in patients with primary premature ovarian failure. Clin. Res. Cardiol..

[81] Langrish J.P., Mills N.L., Bath L.E., Warner P., Webb D.J., Kelnar C.J., Critchley H.O., Newby D.E., Wallace W.H. (2009). Cardiovascular effects of physiological and standard sex steroid replacement regimens in premature ovarian failure. Hypertension.

[82] Aksoy M.N., Akdemir N., Kilic H., Yilmaz S., Akdemir R., Gunduz H. (2017). Pulse wave velocity and myocardial performance index in premature ovarian insufficiency. Scand. Cardiovasc. J..

[83] Blumel J.E., Mezones-Holguin E., Chedraui P., Soto-Becerra P., Arteaga E., Vallejo M.S. (2022). Is premature ovarian insufficiency associated with mortality? A three-decade follow-up cohort. Maturitas.

[84] Van Lennep J.E.R., Heida K.Y., Bots M.L., Hoek A., on behalf of the collaborators of the Dutch Multidisciplinary Guideline Development Group on Cardiovascular Risk Management after Reproductive Disorders (2016). Cardiovascular disease risk in women with premature ovarian insufficiency: A systematic review and meta-analysis. Eur. J. Prev. Cardiol..

